# Values of COVID-19 Self-Testing among Urban and Rural South Africans: A Cross-Sectional Survey

**DOI:** 10.1016/j.pmedr.2023.102114

**Published:** 2023-01-18

**Authors:** Amanda N. Brumwell, Gbotemi B. Babatunde, Michael W. Wilson, Karl le Roux, Monique M. Marks, Jamila K. Adam, Elena Ivanova, Deepshikha Batheja, Srishti Goel, Sonjelle Shilton, Guillermo Z. Martínez-Pérez

**Affiliations:** a Advance Access & Delivery South Africa (AA&D SA), Durban, South Africa; b Department of Health Behavior, University of North Carolina Gillings School of Global Public Health, Chapel Hill, NC, USA; c Zithulele Hospital, Zithulele, Mqanduli, South Africa; d Durban University of Technology, Durban, South Africa; e FIND, the global alliance for diagnostics, Geneva, Switzerland; f Centre for Disease Dynamics, Economics and Policy, India

**Keywords:** COVID-19, Home diagnostics, South Africa, SARS-CoV-2 testing, Self-testing, Survey

## Abstract

Self-testing for COVID-19 may be a preferable strategy for identifying SARS-CoV-2 infection among populations in low- and middle-income settings. To determine South Africans’ values related to COVID-19 self-testing should it become widely available, a cross-sectional survey was administered in Durban, KwaZulu-Natal Province and the King Sabata Dalindyebo sub-district of the Eastern Cape.

A 35-question survey was administered to 531 participants (268 female) in one urban and one rural setting of South Africa. Survey participants were randomly selected by household in the rural setting, while in the urban setting participants were approached in randomly selected public places. The survey assessed participants’ likelihood of using and willingness to pay for a COVID-19 self-test and actions they would take following a COVID-19 self-test. The results were analysed using descriptive statistics and bivariate and multivariate regression.

Overall, 93.03% of participants supported COVID-19 self-testing, 61.62% of participants were willing to pay for self-testing, and 90.15% indicated they would communicate their results if they tested positive. Rural participants were more positively associated with each of these outcomes compared with urban-based participants. Should they test positive, most participants said they would: go in-person to a health facility for counselling (76.45%), self-isolate (95.85%), notify close contacts (97.74%), and inform their employer (95.14%).

COVID-19 self-testing was a preferable option for most participants, although this varied with setting and demographic characteristics. Self-testing may overcome barriers to care for South Africans, but to achieve this, policies for self-testing and delivery methods must not exacerbate individuals’ underlying economic vulnerabilities.

## 1 Introduction

Nearly three years after the COVID-19 pandemic was declared (Cucinotta & Vanelli, 2020) much of the world has transitioned from responding to COVID-19 as a public health emergency towards managing the disease as endemic. In South Africa a state of disaster was declared on 15 March 2020, which precipitated mass community testing in an effort to halt transmission of the virus.

The South African government deployed cadres of lay healthcare workers in community-based settings to provide professional rapid COVID-19 antigen-detection tests (RADTs) (Baxter et al., 2021). However, this strategy did not sufficiently meet the population’s needs for COVID-19 testing. South Africa's health system had long experienced structural and resource challenges that worsen essential medical care delivery (Mayosi & Benatar, 2014). These conditions limited the reach and efficacy of the health department’s widespread screening campaigns.

Self-testing is recognised as a useful tool to complement COVID-19 control strategies by the African CDC, the World Health Organization (WHO), and the American Food and Drug Administration (FDA) (Africa Centres for Disease Control and Prevention, 2022; US Food & Drug Administration, 2022, World Health Organization, 2022). COVID-19 self-tests are almost as accurate as professional RADTs, and their acceptability has been demonstrated in other settings, including other low- and middle-income countries including Indonesia, Kenya, and Nigeria and upper-middle income countries including Brazil and Greece (Chabeda et al., 2022, Harmon et al., 2021, Manguro et al., 2022, Martínez-Pérez and Schirmer, 2022, Thomas et al., 2022, Goggolidou et al., 2021, Undelikwo et al., 2022). However, to our knowledge, there has been no study to date regarding values of COVID-19 self-testing among the general population of the southern African region. Self-testing using RADTs may complement existing, community-based efforts to bridge the gap in the South African population’s needs for COVID-19 testing, especially since the government repealed the COVID-19 regulations as of 22 June 2022 (South African Government, 2022b).

In South Africa, self-tests are already used to diagnose HIV infection and for monitoring other conditions such as pregnancy, blood glucose levels, and drug use, although kit availability varies widely, and purchase is often cost-prohibitive (Pillay & Aldous, 2016; South African Government, 2022a; Venter et al., 2017). Given the high acceptability of relatively novel self-testing methods such as those for HIV, South African communities may exhibit similar acceptance towards COVID-19 self-testing (Knight et al., 2017, Lippman et al., 2018). However, the public’s attitudes towards COVID-19 self-testing and preferences for its delivery must be assessed, to provide effective education and acceptable delivery strategies.

To this end, a survey was administered among the general population in Durban, KwaZulu-Natal Province and the rural King Sabata Dalindyebo (KSD) sub-district, Eastern Cape Province, to gauge these communities’ values of and attitudes towards SARS-CoV-2 self-testing. Other specific objectives were to understand the predictors of likelihood of using self-testing, willingness to pay for self-testing, and adherence to recommended actions following a positive result.

## 2 Methods

### 2.1 Design, population, and sites

This study employed a cross-sectional survey design. It was conducted in September 2021, in two provinces in South Africa: Durban, in KwaZulu-Natal province, representing urban and *peri*-urban areas, while the catchment area of Zithulele District Hospital, in KSD municipality of the Eastern Cape province, represented the rural area.

Sample size calculations, performed separately for each site, estimated that at least 196 survey respondents in Durban and KSD, respectively, were necessary to have a 95 % confidence level that the real value (of likelihood to use COVID-19 self-testing) was within ± 7 % of the measured value.

### 2.2 Sampling and enrolment of survey respondents

The surveys were initially planned to be household-based. However, following incidents of violence and mass looting in Durban in July 2021 (Kalina, 2021), the surveys in Durban were administered in community spaces (e.g. taxi ranks, shops, malls) to ensure the safety of surveyors and respondents. The rural KSD area was unaffected by violence, so the surveys remained household-based.

This study employed a five-pronged sampling process. First, the boundaries for each site were determined using Google MyMaps, with the resulting maps divided into 40 numbered areas of similar size. Second, the list of numbered areas for each site was rearranged using the random list generator, Random.org®. The first 14 sites in each rearranged list were selected as survey areas. Third, these 14 areas on each list were assigned to a survey shift. Fourth, the recruiting points were selected in MyMaps. In the rural area, 21 households were randomly chosen, while in the urban areas public spaces or community gathering venues such as post offices, health centres, or supermarkets were selected. Fifth, the survey respondents were randomly chosen in the households and community gathering venues by the surveyors.

### 2.3 Data collection, processing, and analysis

A 35-item structured questionnaire was employed, based on an adapted version of an instrument previously used to assess values and preferences for hepatitis C virus self-testing (Martínez-Pérez et al., 2021). It included four main sections: socio-demographics; perception of risk of COVID-19 and previous experiences with COVID-19 testing; likelihood to use and willingness to pay for a SARS-CoV-2 self-test; and likely actions following self-testing. The questionnaire was written in English, re-tested during the training of surveyors. The questionnaire was translated into isiXhosa for use in KSD.

Each surveyor completed the questionnaire for all respondents using the KoBoCollect® app installed on tablet computers; responses were immediately submitted to KoBoToolbox®. All submitted data were anonymous.

Data were exported into MS-Excel, merged, and cleaned. STATA was used to run descriptive and bivariate and multivariate analyses. Bivariate and multivariate regression analyses were performed for each of the three outcomes: likelihood to use a SARS-CoV-2 self-test; willingness to pay for a self-test device; likelihood to comply with recommended actions following a positive self-test result (i.e., communicate the result, warn close contacts, self-isolate, request post-test counselling). The variables found by the bivariate analyses as significantly associated with the outcomes at a P < 0.05 were considered for the multivariate analyses. An ordinal logistic regression model was used to identify associations between binary responses to outcomes on likelihood to use a self‐test (likely/unlikely), willingness to pay (any amount/no amount), and potential predictors. An ordinary least squares (OLS) regression was used to identify potential predictors of compliance with the four expected recommended actions following a positive result.

### 2.4 Ethics

All survey respondents gave informed consent. Each respondent received face masks and hand sanitiser as a token for their participation. This research received ethical clearance from the Durban University of Technology Institutional Research Ethics Committee (IREC 165/21).

## 3 Results

### 3.1 Respondents’ characteristics

531 individuals participated in this survey. Of these, 268 (50.47 %) were female, 274 (51.60 %) of the total were respondents in the rural KSD (Table 1). The median age of respondents was 37 years (interquartile range (IQR) = 24).

**Table 1:** Age, education, and employment status of survey participants.

	Rural		Urban		Subtotal (male and female)		Subtotal (rural and urban)		Total
	Female (n = 164)	Male (n=110)	Female (n=104)	Male (n=153)	Rural (n=274)	Urban (n=257)	Female (n=268)	Male (n=263)	(n=531)
**Age**									
Median age (years)	40	41.5	35	34	40.5	34	38	36	37
Interquartile Range	29.5	34	18	17	32	17	25.5	23	24
**Age group**									
18–35	65 (39.63%)	47 (42.72%)	53 (50.96%)	83 (54.24%)	112 (40.87%)	136 (52.91%)	118 (44.02%)	130 (49.42%)	248 (46.70%)
36–55	55 (33.53%)	27 (24.54%)	36 (34.61%)	56 (36.60%)	82 (29.92%)	92 (35.79%)	91 (33.95%)	83 (31.55%)	174 (32.76%)
56 and over	44 (26.82%)	36 (32.72%)	15 (14.42%)	14 (9.15%)	80 (29.19%)	29 (11.28%)	59 (22.01%)	50 (19.01%)	109 (20.52%)
**Education**									
None	22 (13.41%)	11 (10.00%)	0 (0.00%)	2 (1.31%)	33 (12.04%)	2 (0.78%)	22 (8.27%)	13 (4.96%)	35 (6.62%)
Primary	44 (26.82%)	42 (38.18%)	8 (7.84%)	5 (3.28%)	86 (31.38%)	13 (5.11%)	52 (19.54%)	47 (17.93%)	99 (18.75%)
Secondary	84 (51.21%)	54 (49.09%)	53 (51.96%)	93 (61.18%)	138 (50.36%)	146 (57.48%)	137 (51.50%)	147 (56.10%)	284 (53.78%)
College/vocational training	10 (6.09%)	3 (2.72%)	16 (15.68%)	20 (13.15%)	13 (4.74%)	36 (14.17%)	26 (9.77%)	23 (8.77%)	49 (9.28%)
University Degree/Bachelor's	4 (2.43%)	0 (0.00%)	21 (20.58%)	30 (19.73%)	4 (1.45%)	51 (20.07%)	25 (9.39%)	30 (11.45%)	55 (10.41%)
Postgraduate/Master's	0 (0.00%)	0 (0.00%)	4 (3.92%)	2 (1.31%)	0 (0.00%)	6 (2.36%)	4 (1.50%)	2 (0.76%)	6 (1.13%)
Other	0 (0.00%)	0 (0.00%)	0 (0.00%)	0 (0.00%)	0 (0.00%)	0 (0.00%)	0 (0.00%)	0 (0.00%)	0 (0.00%)
**Employment**									
Unemployed	112 (68.29%)	70 (63.63%)	25 (24.03%)	33 (21.56%)	182 (66.42%)	58 (22.56%)	137 (51.11%)	103 (39.16%)	240 (45.19%)
Student	11 (6.70%)	4 (3.63%)	11 (10.57%)	10 (6.53%)	15 (5.47%)	21 (8.17%)	22 (8.20%)	14 (5.32%)	36 (6.77%)
Employed, part-time	11 (6.70%)	6 (5.45%)	12 (11.53%)	24 (15.68%)	17 (6.20%)	36 (14.00%)	23 (8.58%)	30 (11.40%)	53 (9.98%)
Employed, full-time	7 (4.26%)	8 (7.27%)	45 (43.26%)	58 (37.90%)	15 (5.47%)	103 (40.07%)	52 (19.40%)	66 (25.09%)	118 (22.22%)
Self-employed, part-time	2 (1.21%)	2 (1.81%)	2 (1.92%)	4 (2.61%)	4 (1.45%)	6 (2.33%)	4 (1.49%)	6 (2.28%)	10 (1.88%)
Self-employed, full-time	0 (0.00%)	2 (1.81%)	4 (3.84%)	19 (12.41%)	2 (0.72%)	23 (8.94%)	4 (1.49%)	21 (7.98%)	25 (4.70%)
Retired on a pension	21 (12.80%)	18 (16.36%)	5 (4.80%)	5 (3.26%)	39 (14.23%)	10 (3.89%)	26 (9.70%)	23 (8.74%)	49 (9.22%)

Overall, 53.79 % of respondents had completed at least secondary education, 9.28 % had completed college or vocational training, and 10.42 % had completed a university degree or bachelor’s-level education. Higher levels of education were reported among Durban-based respondents.

In total, 45.20 % of respondents were reportedly unemployed, while 22.22 % were employed full-time. Rural respondents reported higher levels of unemployment (66.42 %) compared with urban respondents (22.56 %), and urban individuals reported higher rates of full-time employment compared with their rural counterparts (40.07 % versus 5.47 %, respectively).

### 3.2 COVID-19 testing experience and risk perception

In total, 75.51 % of respondents reported never having tested for COVID-19, and 28.44 % respondents reported experiencing at least one instance when they felt they needed a COVID-19 test but could not access testing (Table 2). The proportion of individuals having never tested was higher among rural respondents (89.05 %) than urban respondents (61.08 %). Among the 24.49 % of respondents who reported having tested for COVID-19 at least once, 61.24 % considered their previous COVID-19 testing experience as “convenient” or “very convenient”.

**Table 2:** Risk perception and experience of COVID-19.

**Survey Question**	**Percentage (%) responding in the affirmative (i.e. “Yes”)**								
	**Rural**		**Urban**		**Total (Male and Female)**		**Total(Rural and Urban)**		
	**Female (n = 164)**	**Male (n = 110)**	**Female (n = 104)**	**Male (n = 153)**	**Rural(n = 274)**	**Urban(n = 257)**	**Female (n = 268)**	**Male (n = 263)**	**Total(n = 531)**
**How do you perceive your risk of getting COVID-19 today?**									
Low risk	19 (18.29 %)	19 (17.27 %)	7 (15.38 %)	12 (18.30 %)	38 (17.88 %)	19 (17.12 %)	26 (17.16 %)	31 (17.87 %)	57 (17.51 %)
Mild risk	30 (14.63 %)	19 (12.72 %)	16 (8.65 %)	28 (15.68 %)	49 (13.86 %)	44 (12.84 %)	46 (12.31 %)	47 (14.44 %)	93 (13.37 %)
Moderate risk	24 (15.24 %)	14 (18.18 %)	9 (9.61 %)	24 (14.37 %)	38 (16.42 %)	33 (12.45 %)	33 (13.05 %)	38 (15.96 %)	71 (14.50 %)
High risk	25 (40.24 %)	20 (34.54 %)	10 (59.61 %)	22 (43.79 %)	45 (37.95 %)	32 (50.19 %)	35 (47.76 %)	42 (39.92 %)	77 (43.87 %)
**Are there people (e.g., elders, people with chronic diseases) in your household that are athigh-risk of getting very sick from COVID-19?**									
Children	0 (0.00 %)	2 (1.81 %)	9 (8.65 %)	18 (11.76 %)	2 (0.72 %)	27 (10.50 %)	9 (3.35 %)	20 (7.60 %)	29 (5.46 %)
Children and elders	2 (1.21%)	0 (0.00%)	14 (13.46%)	26 (16.99%)	2 (0.72%)	40 (15.56%)	16 (5.97%)	26 (9.88%)	42 (7.90%)
Children and elders and chronic diseases	0 (0.00%)	0 (0.00%)	1 (0.96%)	4 (2.61%)	0 (0.00%)	5 (1.94%)	1 (0.37%)	4 (1.52%)	5 (0.94%)
Elders only	20 (12.19%)	12 (10.90%)	25 (24.03%)	28 (18.30%)	32 (11.67%)	53 (20.62%)	45 (16.79%)	40 (15.20%)	85 (16.00%)
Elders and chronic diseases	9 (5.48%)	6 (5.45%)	1 (0.96%)	3 (1.96%)	15 (5.47%)	4 (1.55%)	10 (3.73%)	9 (3.42%)	19 (3.57%)
Chronic diseases only	52 (31.70%)	38 (34.54%)	11 (10.57%)	12 (7.84%)	90 (32.84%)	23 (8.94%)	63 (23.50%)	50 (19.01%)	113 (21.28%)
Children and chronic diseases	0 (0.00%)	0 (0.00%)	1 (0.96%)	2 (1.30%)	0 (0.00%)	3 (1.16%)	1 (0.37%)	2 (0.76%)	3 (0.56%)
**Have you ever had COVID-19?**									
Yes, confirmed by a test	3 (1.82 %)	2 (1.81 %)	17 (16.34 %)	27 (17.76 %)	5 (1.82 %)	44 (17.18 %)	20 (7.46 %)	29 (11.06 %)	49 (9.24 %)
Yes, confirmed by a healthcare worker (no test involved)	0 (0.00 %)	0 (0.00 %)	0 (0.00 %)	5 (3.28 %)	0 (0.00 %)	5 (1.95 %)	0 (0.00 %)	5 (1.90 %)	5 (0.94 %)
I think so, but not confirmed	0 (0.00 %)	0 (0.00 %)	1 (0.96 %)	1 (0.65 %)	0 (0.00 %)	2 (0.78 %)	1 (0.37 %)	1 (0.38 %)	2 (0.37 %)
No, never	161 (98.17 %)	108 (98.18 %)	82 (78.84 %)	111 (73.02 %)	269 (98.17 %)	193 (75.39 %)	243 (90.67 %	219 (83.58 %)	462 (87.16 %)
Not sure/Do not know	0 (0.00 %)	0 (0.00 %)	4 (3.84 %)	8 (5.26 %)	0 (0.00 %)	12 (4.68 %)	4 (1.49 %)	8 (3.05 %)	12 (2.26 %)
**If you had a test that confirmed that you were positive for COVID-19, did you self-isolate?**									
Yes	2 (66.66 %)	2 (100.00 %)	15 (93.75 %)	25 (92.59 %)	4 (80.00 %)	40 (93.02 %)	17 (89.47 %)	27 (93.10 %)	44 (91.66 %)
No	1(33.33 %)	0 (0.00 %)	1 (6.25 %)	2 (7.40 %)	1 (20.00 %)	3 (6.97 %)	2 (10.52 %)	2 (6.89 %)	4 (8.33 %)
**No. of times have you felt that you needed testing for COVID-19 but you could NOT access testing**									
Never	114 (69.51 %)	85 (77.27 %)	64 (61.53 %)	100 (65.35 %)	199 (72.62 %)	164 (63.81 %)	178 (62.23 %)	185 (70.34 %)	363 (68.36 %)
Not sure/cannot remember	0 (0.00 %)	0 (0.00 %)	8 (0.96 %)	9 (0.00 %)	0 (0.00 %)	17 (0.38 %)	8 (0.34 %)	9 (0.00 %)	17 (0.18 %)
At least once	50 (9.14 %)	25 (13.63 %)	32 (41.34 %)	44 (36.60 %)	75 (10.94 %)	76 (38.52 %)	82 (20.27 %)	69 (26.99 %)	151 (24.29 %)
**Ever tested for COVID-19**									
Never	149 (90.85 %)	95 (86.36 %)	60 (57.69 %)	97 (63.39 %)	244 (89.05 %)	157 (61.08 %)	209 (73.07 %)	192 (73.00 %)	401 (75.51 %)
Not sure/cannot remember	0 (0.00 %)	0 (0.00 %)	1 (0.96 %)	0 (0.00 %)	0 (0.00 %)	1 (0.38 %)	1 (0.34 %)	0 (0.00 %)	1 (0.18 %)
At least once	15 (9.14 %)	15 (13.63 %)	43 (41.34 %)	56 (36.60 %)	30 (10.94 %)	99 (38.52 %)	58 (20.27 %)	71 (26.99 %)	129 (24.29 %)
**How convenient was your last experience receiving a test for COVID-19?**									
Very convenient	1 (6.66 %)	3 (20.00 %)	16 (37.20 %)	25 (44.64 %)	4 (13.33 %)	41 (41.41 %)	17 (29.31 %)	28 (39.43 %)	45 (34.88 %)
Convenient	4 (26.66 %)	5 (33.33 %)	13 (30.23 %)	12 (21.42 %)	9 (30.00 %)	25 (25.25 %)	17 (29.31 %)	17 (23.94 %)	34 (26.35 %)
Neutral	5 (33.33 %)	4 (26.66 %)	3 (6.97 %)	3 (5.35 %)	9 (30.00 %)	6 (6.06 %)	8 (13.79 %)	7 (9.85 %)	15 (11.62 %)
Inconvenient	6 (40.00 %)	2 (13.33 %)	4 (9.30 %)	7 (12.50 %)	8 (26.66 %)	11 (11.11 %)	10 (17.24 %)	9 (12.67 %)	19 (14.72 %)
Very inconvenient	0 (0.00 %)	0 (0.00 %)	7 (16.27 %)	9 (16.07 %)	0 (0.00 %)	16 (16.16 %)	7 (12.06 %)	9 (12.67 %)	16 (12.40 %)

9.25 % reported having previously had test-confirmed COVID-19 disease; 89.80 % of these were based in Durban. Most individuals (91.67 %) with test-confirmed COVID-19 reported self-isolating.

Overall, 50.19 % and 37.95 % of urban and rural respondents, respectively, considered themselves to be at high risk of becoming sick with COVID-19 (Table 2). Respondents reported living with individuals with chronic disease (21.28 %) and elders (16.01 %), two groups at risk of severe COVID-19.

### 3.3 Knowledge, acceptability, and likelihood of COVID-19 self-testing

Regarding knowledge of self-testing kits for conditions other than COVID-19, respondents reported awareness of devices to self-test for HIV (47.80 %), pregnancy (57.80 %), diabetes (31.30 %), and hypertension (21.30 %) (Table 3). Only three respondents, all male, knew of COVID-19 self-tests.

**Table 3:** Knowledge, awareness, and acceptability of COVID-19 self-testing.

**Survey question**	**Percentage (%) responding in the affirmative (i.e. “Yes”)**								
	**Rural**		**Urban**		**Total (male and female)**		**Total** **(rural and urban)**		
	**Female (n = 164)**	**Male (n = 110)**	**Female (n = 104)**	**Male (n = 153)**	**Rural(n = 274)**	**Urban(n = 257)**	**Female (n = 268)**	**Male (n = 263)**	**Total(n = 531)**
**Awareness of self-testing devices**									
HIV	50 (30.48 %)	39 (35.45 %)	59 (56.73 %)	63 (41.17 %)	89 (32.48 %)	122 (47.47 %)	109 (38.11 %)	102 (38.78 %)	211 (39.73 %)
Malaria	0 (0.00 %)	0 (0.00 %)	2 (1.92 %)	5 (3.26 %)	0 (0.00 %)	7 (2.72 %)	2 (0.69 %)	5 (1.90 %)	7 (1.31 %)
Syphilis	0 (0.00 %)	0 (0.00 %	2 (1.92 %)	2 (1.30 %)	0 (0.00 %)	4 (1.55 %)	2 (0.69 %)	2 (0.76 %)	4 (0.75 %)
Ulcer (*Helicobacter pylori*)	0 (0.00 %)	0 (0.00 %)	0 (0.00 %)	1 (0.65 %)	0 (0.00 %)	1 (0.38 %)	0 (0.00 %)	1 (0.38 %)	1 (0.18 %)
COVID-19	0 (0.00 %)	1 (0.90 %)	0 (0.00 %)	2 (1.30 %)	1 (0.36 %)	2 (0.77 %)	0 (0.00 %)	3 (1.14 %)	3 (0.56 %)
Hepatitis C	0 (0.00 %)	0 (0.00 %)	1 (0.96 %)	0 (0.00 %)	0 (0.00 %)	1 (0.38 %)	1 (0.34 %)	0 (0.00 %)	1 (0.18 %)
Hypertension	29 (17.68 %)	19 (17.27 %)	21 (20.19 %)	25 (16.33 %	48 (17.51 %)	46 (17.89 %)	50 (17.48 %)	44 (16.73 %)	94 (17.70 %)
Diabetes/glycaemia	33 (20.12 %	33 (30.00 %)	33 (31.73 %)	39 (25.49 %)	66 (24.08 %)	72 (28.01 %)	66 (23.07 %)	72 (27.37 %)	138 (25.98 %)
Pregnancy	109 (66.46 %)	19 (17.27 %)	62 (59.61 %)	65 (42.48 %)	128 (46.71 %)	127 (49.41 %)	171 (59.79 %)	84 (31.93 %)	255 (48.02 %)
Substances(alcohol, cocaine, marijuana etc.)	0 (0.00 %)	2 (1.81 %)	8 (7.69 %)	17 (11.11 %)	2 (0.72 %)	25 (9.72 %)	8 (2.79 %)	19 (7.22 %)	27 (5.08 %)
**Agreement with the concept of people being able to self-test at home on their own for COVID-19 disease**									
Yes	159 (96.95 %)	108 (98.18 %)	94 (90.38 %)	133 (86.92 %)	267 (97.44 %)	227 (88.32 %)	253 (88.46 %)	241 (91.63 %)	494 (93.03 %)
No	2 (1.21 %)	2 (1.81 %)	5 (4.80 %)	13 (8.49 %)	4 (1.45 %)	18 (7.00 %)	7 (2.44 %)	15 (5.70 %)	22 (4.14 %)
Not sure/cannot say	3 (1.82 %)	0 (0.00 %)	5 (4.80 %)	7 (4.57 %)	3 (1.09 %)	12 (4.66 %)	8 (2.79 %)	7 (2.66 %)	15 (2.82 %)
**Likelihood of using a COVID-19 self-test, if COVID-19 self-tests were available in this country, and you felt you needed to test for COVID-19**									
Very unlikely	2 (1.21 %)	2 (1.81 %)	5 (4.80 %)	11 (7.18 %)	4 (1.45 %)	16 (6.22 %)	7 (2.44 %)	13 (4.94 %)	20 (3.76 %)
Unlikely	1 (0.60 %)	1 (0.90 %)	1 (0.96 %)	4 (2.61 %)	2 (0.72 %)	5 (1.94 %)	2 (0.69 %)	5 (1.90 %)	7 (1.31 %)
Neutral	1 (0.60 %)	1 (0.90 %)	9 (8.65 %)	13 (8.49 %)	2 (0.72 %)	22 (8.56 %)	10 (3.49 %)	14 (5.32 %)	24 (4.51 %)
Likely	21 (12.80 %)	13 (11.81 %)	15 (14.42 %)	38 (24.83 %)	34 (12.40 %)	53 (20.62 %)	36 (12.58 %)	51 (19.39 %)	87 (16.38 %)
Very Likely	139 (84.75 %)	93 (84.54 %)	74 (71.15 %)	87 (56.86 %)	232 (84.67 %)	161 (62.64 %)	213 (74.47 %)	180 (68.44 %)	393 (74.01 %)
**Willingness to pay**									
Willing to pay if self-tests were not provided for free by health authorities	37 (77.30 %)	34 (69.09 %)	50 (51.92 %)	82 (46.05 %)	71 (73.99 %)	132 (48.43 %)	87 (67.41 %)	116 (55.72 %)	203 (61.62 %)

More rural respondents (97.45 %) supported the concept of home self-testing for COVID-19 than urban respondents (88.33 %). Rural respondents more commonly (97.08 %) reported that they would be likely or very likely to use COVID-19 self-testing compared with urban respondents (83.27 %).

The primary enablers for respondents’ likelihood of using COVID-19 self-testing included that self-testing would require less time compared with waiting for facility-based testing (56.28 %), would provide results more rapidly than other forms of testing (47.37 %), and would save respondents money on transportation (44.33 %) (Table 4). Among respondents who indicated they would be unlikely or very unlikely to use COVID-19 self-testing, the most common deterrents mentioned were: the fear of long wait times to receive results (26.86 %); fears that one would need to travel to a facility anyway for care or confirmatory testing (19.40 %); and concerns that care would be unavailable in the event of a positive self-test (17.91 %) (Table 5).

**Table 4:** Advantages of COVID-19 self-testing.

	**Percentage (%) responding in the affirmative (i.e. “Yes”)**								
	**Rural**		**Urban**		**Total (male and female)**		**Total (rural and urban)**		**Total**
	**Female (n = 164)**	**Male (n = 110)**	**Female (n = 104)**	**Male (n = 153)**	**Rural (n = 274)**	**Urban (n = 257)**	**Female (n = 268)**	**Male (n = 263)**	**Total(n = 531)**
**Factors that would determine likelihood of using a COVID-19 self-test**									
It will allow me to know my test results faster	49 (32.45 %)	43 (40.18 %)	59 (61.45 %)	83 (59.28 %)	92 (35.65 %)	142 (60.16 %)	108 (43.72 %)	126 (51.01 %)	234 (47.36 %)
It would allow me to request treatment faster/before I get too ill	28 (18.54 %)	28 (26.16 %)	29 (30.20 %)	40 (20.71 %)	56 (21.70 %)	69 (29.23 %)	57 (23.07 %)	68 (27.53 %)	125 (25.30 %)
It will allow me to make the test in privacy (and keep my results confidential)	19 (12.58 %)	14 (13.08 %)	43 (44.79 %)	48 (30.71 %)	33 (12.79 %)	91 (38.55 %)	62 (25.10 %)	62 (25.10 %)	124 (25.10 %)
It will allow me to calm my anxiety/fears about the disease	5 (3.31 %)	5 (4.67 %)	24 (25.00 %)	34 (17.14 %)	10 (3.87 %)	58 (24.57 %)	29 (11.74 %)	39 (15.78 %)	68 (13.76 %)
It will be less painful (or pain-free) than a clinic/lab test	16 (10.59 %)	14 (13.08 %)	20 (20.83 %)	30 (14.28 %)	30 (11.62 %)	50 (21.18 %)	36 (14.57 %)	44 (17.81 %)	80 (16.19 %)
It will save me time for travelling to/waiting in a clinic/lab	100 (66.22 %)	70 (65.42 %)	46 (47.91 %)	62 (32.85 %)	170 (65.89 %)	108 (45.76 %)	146 (59.10 %)	132 (53.44 %)	278 (56.27 %)
It will save me money for travelling to/testing in a clinic/lab	78 (51.65 %)	52 (48.59 %)	41 (42.70 %)	48 (29.28 %)	130 (50.38 %)	89 (37.71 %)	119 (48.17 %)	100 (40.48 %)	219 (44.33 %)
It will help me to not deal with healthcare staff	9 (5.96 %)	8 (7.47 %)	28 (29.16 %)	32 (20.00 %)	17 (6.58 %)	60 (25.42 %)	37 (14.97 %)	40 (16.19 %)	77 (15.58 %)
It will help me not to expose myself to COVID-19 in any testing site	19 (12.58 %)	10 (9.34 %)	27 (28.12 %)	32 (19.28 %)	29 (11.24 %)	59 (25.00 %)	46 (18.62 %)	42 (17.00 %)	88 (17.81 %)
I will not risk losing my job/wages (should the self-test be positive)	0 (0.00 %)	0 (0.00 %)	1 (1.04 %)	6 (0.71 %)	0 (0.00 %)	7 (2.96 %)	1 (0.40 %)	6 (2.42 %)	7 (1.41 %)
It will be cheaper	7 (4.63 %)	3 (2.80 %)	12 (12.50 %)	15 (8.57 %)	10 (3.87 %)	27 (11.44 %)	19 (7.69 %)	18 (7.28 %)	37 (7.48 %)
It will be useful for work/school testing (integrate testing out of labs/clinics)	2 (1.32 %)	0 (0.00 %)	4 (4.16 %)	3 (2.85 %)	2 (0.77 %)	7 (2.96 %)	6 (2.42 %)	3 (1.21 %)	9 (1.82 %)
It will not be difficult to use/understand the instructions (it will be easy)	0 (0.00 %)	1 (0.93 %)	3 (3.12 %)	4 (2.14 %)	1 (0.38 %)	7 (2.96 %)	3 (1.21 %)	5 (2.02 %)	8 (1.61 %)
I will trust the result (It will be accurate/precise)	2 (1.32 %)	0 (0.00 %)	5 (5.20 %)	8 (3.57 %)	2 (0.77 %)	13 (5.50 %)	7 (2.83 %)	8 (3.23 %)	15 (3.03 %)

**Table 5:** Disadvantages of COVID-19 self-testing.

	**Percentage (%) responding in the affirmative (i.e. “Yes”)**								
	**Rural**		**Urban**		**Total (male and female)**		**Total (rural and urban)**		**Total**
	**Female (n = 164)**	**Male (n = 110)**	**Female (n = 104)**	**Male (n = 153)**	**Rural (n = 274)**	**Urban (n = 257)**	**Female (n = 268)**	**Male (n = 263)**	**Total** **(n = 531)**
I will have to wait too long to know the result	4 (28.57 %)	3 (60.00 %)	4 (20.00 %)	7 (25.00 %)	7 (36.84 %)	11 (22.91 %)	8 (23.52 %)	10 (30.30 %)	18 (26.86 %)
I will not be able to access/request treatment afterwards (if positive)	1 (7.14 %)	0 (0.00 %)	2 (10.00 %)	9 (32.14 %)	1 (5.26 %)	11 (22.91 %)	3 (8.82 %)	9 (27.27 %)	12 (17.91 %)
I will not have a place to make the test in privacy (and keep my results confidential)	0 (0.00 %)	0 (0.00 %)	1 (5.00 %)	4 (14.28 %)	0 (0.00 %)	5 (10.41 %)	1 (2.94 %)	4 (12.12 %)	5 (7.46 %)
It will increase my anxiety/fears about the disease	0 (0.00 %)	1 (20.00 %)	2 (10.00 %)	5 (17.85 %)	1 (5.26 %)	7 (14.58 %)	2 (5.88 %)	6 (18.18 %)	8 (11.94 %)
It will be more painful (or not pain-free) than a clinic/lab test	1 (7.14 %)	1 (20.00 %)	3 (15.00 %)	7 (25.00 %)	2 (10.52 %)	10 (20.83 %)	4 (11.76 %)	8 (24.24 %)	12 (17.91 %)
I will better use a professional test in a lab/clinic	0 (0.00 %)	2 (40.00 %)	3 (15.00 %)	5 (17.85 %)	2 (10.52 %)	8 (16.66 %)	3 (8.82 %)	7 (21.21 %)	10 (14.92 %)
I will have to travel to/wait in a clinic/lab anyway (to confirm, to request care…)	10 (71.42 %)	1 (20.00 %)	2 (10.00 %)	0 (0.00 %)	11 (57.89 %)	2 (4.16 %)	12 (35.29 %)	1 (3.03 %)	13 (19.40 %)
I will have to spend money to travel to/wait in a clinic/lab anyway (to confirm, to request care…)	2 (14.28 %)	1 (20.00 %)	0 (0.00 %)	1 (3.57 %)	3 (15.78 %)	1 (2.08 %)	2 (5.88 %)	2 (6.06 %)	4 (5.97 %)
My partner/family/workmates will not support me to use it	1 (7.14 %)	0 (0.00 %)	0 (0.00 %)	2 (7.14 %)	1 (5.26 %)	2 (4.16 %)	1 (2.94 %)	2 (6.06 %)	3 (4.47 %)
It will be expensive	0 (0.00 %)	1 (20.00 %)	1 (5.00 %)	1 (3.57 %)	1 (5.26 %)	2 (4.16 %)	1 (2.94 %)	2 (6.06 %)	3 (4.47 %)
It will be difficult to use/understand the instructions (e.g., due to technical language)	0 (0.00 %)	1 (20.00 %)	1 (5.00 %)	3 (10.71 %)	1 (5.26 %)	4 (8.33 %)	1 (2.94 %)	4 (12.12 %)	5 (7.46 %)
I will not trust the results (not accurate/precise)	0 (0.00 %)	0 (0.00 %)	2 (10.00 %)	3 (10.71 %)	0 (0.00 %)	5 (10.41 %)	2 (5.88 %)	3 (9.09 %)	5 (7.46 %)
I will not know what to do next with the results	0 (0.00 %)	0 (0.00 %)	1 (5.00 %)	2 (7.14 %)	0 (0.00 %)	3 (6.25 %)	1 (2.94 %)	2 (6.06 %)	3 (4.47 %)

**Fig. 1:**
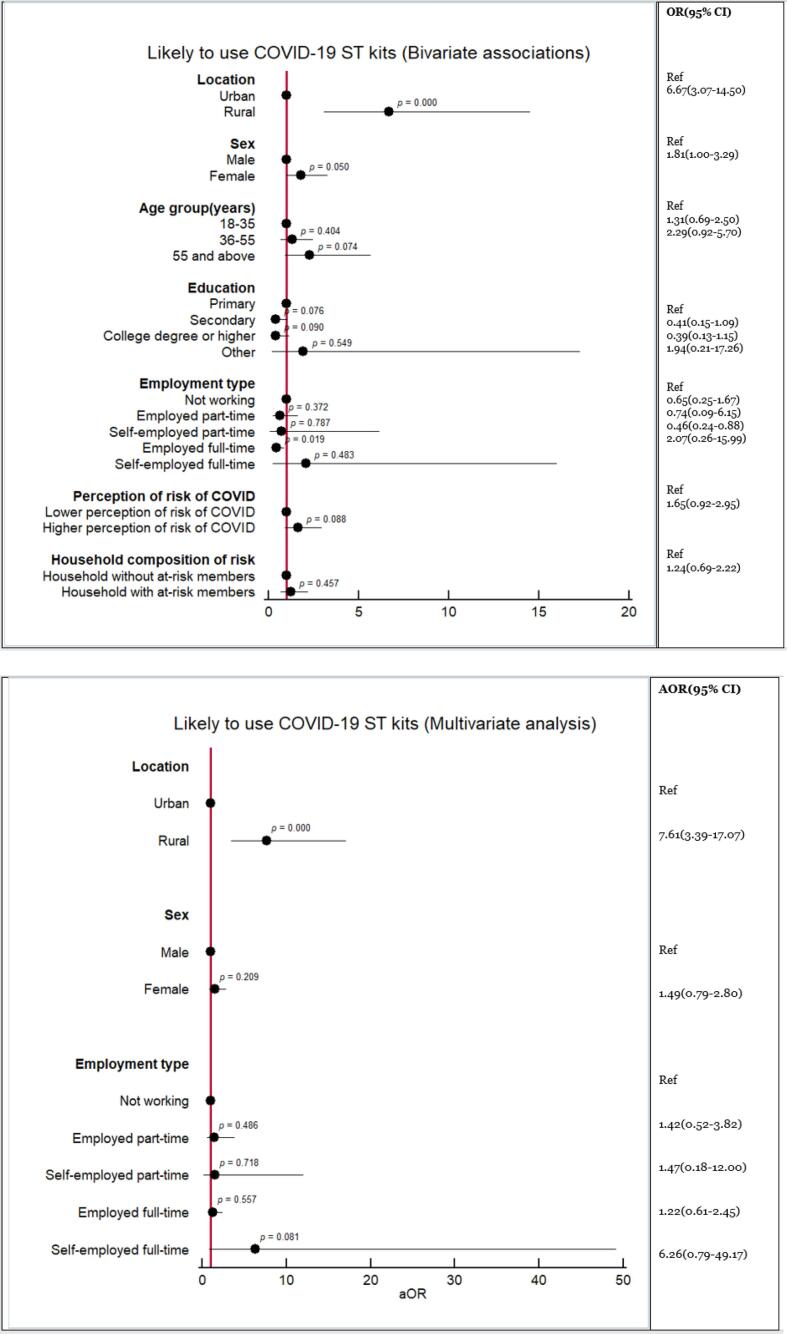
(a) Results of bivariate analysis of the associations between respondents’ characteristics with the likelihood of using a COVID-19 self-test. (b) Results of multivariate analysis of the associations between respondents’ characteristics and the likelihood of using a COVID-19 self-test.

The bivariate analyses (Fig. 1a) suggested that being from a rural area (adjusted odds ratio (AOR) 6.67, 95 % confidence interval (95 %CI): 3.07–14.50, p < 0.000) and being female (AOR 1.81, 95 %CI: 1.00–3.29, p = 0.050) could be predictors of expressing likelihood of using a self-test. Being employed full-time (AOR 0.46, 95 %CI: 0.24–0.88, p = 0.019) was negatively associated with the likelihood of using a self-test. However, the multivariate logistic regression only confirmed that being rural-based was positively associated (AOR 7.61, 95 %CI: 3.39–17.07, p < 0.000) with the likelihood of using COVID-19 self-testing in comparison with being an urban respondent (Fig. 1b).

### 3.4 Willingness to pay for COVID-19 self-testing

Rural respondents were more willing to pay for COVID-19 self-testing (73.99 %) compared with urban respondents (48.44 %) (Table 3). All groups indicated they were willing to pay a nominal fee for self-testing.

**Fig. 2:**
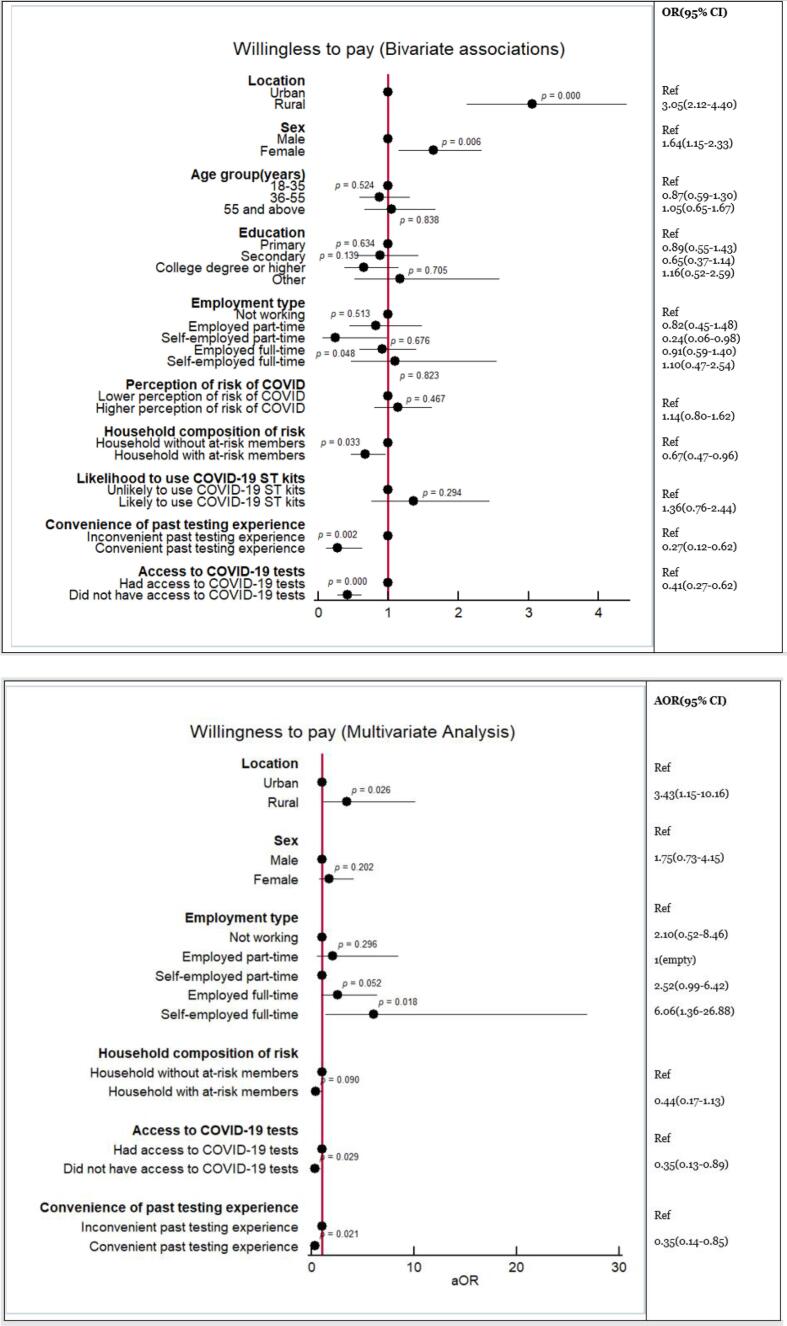
(a) Results of bivariate analysis of the associations of respondents’ characteristics with willingness to pay for COVID-19 self-testing. (b) Results of multivariate analysis of the associations of respondents’ characteristics with willingness to pay for COVID-19 self-testing.

Bivariate analyses suggested that being a rural respondent (AOR 3.05, 95 %CI: 2.12–4.40, p < 0.000) and being female (AOR 1.64, 95 %CI: 1.15–2.33, p = 0.006) could be predictors of willingness to pay, while living in a household with individuals at increased risk of severe COVID-19 (AOR 0.67, 95 %CI: 0.47–0.96, p = 0.033), having had a convenient past testing experience (AOR 0.27, 95 %CI: 0.12–0.62, p = 0.002), or having been unable to access testing when needed (AOR 0.41, 95 %CI: 0.27–0.62, p < 0.000) could be predictors of unwillingness to pay for a self-test (Fig. 2a). The multivariate analyses confirmed that rural residency (AOR 3.43, 95 %CI: 1.15–10.16, p = 0.026) was positively associated with willingness to pay, and that having been unable to access testing (AOR 0.35, 95 %CI: 0.13–0.89, p = 0.029) and having a convenient previous testing experience (AOR 0.35, 95 %CI: 0.14–0.85, p = 0.021) were negatively associated with willingness to pay (Fig. 2b). The multivariate analysis also suggested a positive association with paying for a test if self-employed full-time (AOR 6.06, 95 %CI: 1.36–26.88, p = 0.018), which was not found in the bivariate analyses.

### 3.5 Actions upon self-testing for COVID-19

Rural respondents reported being more likely to communicate a positive COVID-19 self-test result (99.65 %) compared with urban respondents (79.92 %) (Table 6). Preferred options for reporting self-test results included visiting a clinic or hospital (78.41 %), via community health workers (33.71 %), and by phone (33.14 %). Rural respondents indicated a greater willingness (13.14 %) to report results via community health workers. Urban respondents indicated a greater preference for reporting online (18.90 %) or via a pharmacist (14.17 %).

**Table 6:** Actions taken following a positive COVID-19 self-test.

	**Percentage (%) responding in the affirmative (i.e. “Yes”)**								
	Rural		Urban		Total (male and female)		Total (rural and urban)		Total
	Female (n = 164)	Male (n = 110)	Female (n = 104)	Male (n = 153)	Rural (n=274)	Urban (n=257)	Female (n = 268)	Male (n = 263)	(n=531)

**Willingness to communicate results in the event of a positive COVID-19 self-test**									
I wouldn’t communicate/report the result	0 (0.00 %)	1 (0.90 %)	24 (23.07 %)	27 (18.00 %)	1 (0.36 %)	51 (20.07 %)	24 (8.95 %)	28 (10.76 %)	52 (9.84 %)
I would communicate using a medium	164 (100.00 %)	109 (99.09 %)	80 (76.92 %)	123 (82.00 %)	273 (99.63 %)	203 (79.92 %)	244 (91.04 %)	232 (89.23 %)	476 (90.15 %)
**Preferred channels to report a positive test result and access COVID-19 care**									
By going in person to my clinic/hospital (i.e., directly to a healthcare worker)	155 (94.51 %)	106 (96.36 %)	56 (53.84 %)	97 (64.66 %)	261 (95.25 %)	153 (60.23 %)	211 (78.73 %)	203 (78.07 %)	414 (78.40 %)
Through community/village health workers	78 (47.56 %)	59 (53.63 %)	18 (17.30 %)	23 (15.33 %)	137 (50.00 %)	41 (16.14 %)	96 (35.82 %)	82 (31.53 %)	178 (33.71 %)
Through NGO/CSO extension workers	22 (13.41 %)	14 (12.72 %)	6 (5.76 %)	9 (6.00 %)	36 (13.13 %)	15 (5.90 %)	28 (10.44 %)	23 (8.84 %)	51 (9.65 %)
Through phone call (e.g., hotline, toll-free line, COVID line, nearest COVID-19 centre…)	52 (31.70 %)	33 (30.00 %)	44 (42.30 %)	46 (30.66 %)	85 (31.02 %)	90 (35.43 %)	96 (35.82 %)	79 (30.38 %)	175 (33.14 %)
Through the internet (e.g., website, phone application)	1 (0.60 %)	1 (0.90 %)	17 (16.34 %)	31 (20.66 %)	2 (0.72 %)	48 (18.89 %)	18 (6.71 %)	32 (12.30 %)	50 (9.46 %)
Through a pharmacist	4 (2.43 %)	2 (1.81 %)	15 (14.42 %)	21 (14.00 %)	6 (2.18 %)	36 (14.17 %)	19 (7.08 %)	23 (8.84 %)	42 (7.95 %)
Through an employer/boss	3 (1.82 %)	6 (5.45 %)	6 (5.76 %)	10 (6.66 %)	9 (3.28 %)	16 (6.29 %)	9 (3.35 %)	16 (6.15 %)	25 (4.73 %)
Through a teacher/mentor/professor	1 (0.60 %)	2 (1.81 %)	0 (0.00 %)	1 (0.66 %)	3 (1.09 %)	1 (0.39 %)	1 (0.37 %)	3 (1.15 %)	4 (0.75 %)
**If you used a COVID-19 self-test and its result were POSITIVE, would you do the following:**									
**Communicate/report your result to your clinic/hospital and/or to the COVID hotline**									
Yes	161 (98.17 %)	109 (99.09 %)	90 (86.53 %)	133 (86.92 %)	270 (98.54 %)	223 (86.77 %)	251 (93.65 %)	242 (92.01 %)	493 (92.84 %)
No	3 (1.82 %)	1 (0.90 %)	7 (6.73 %)	12 (7.84 %)	4 (1.45 %)	19 (7.39 %)	10 (3.73 %)	13 (4.94 %)	23 (4.33 %)
Not sure/ cannot say	0 (0.00 %)	0 (0.00 %)	7 (6.73 %)	8 (5.22 %)	0 (0.00 %)	15 (5.83 %)	7 (2.61 %)	8 (3.04 %)	15 (2.82 %)
**Go in person to your clinic/hospital to get post-testing counselling from a healthcare professional**									
Yes	158 (96.34 %)	108 (98.18 %)	59 (56.73 %)	81 (52.94 %)	266 (97.08 %)	140 (54.47 %)	217 (80.97 %)	189 (71.86 %)	406 (76.45 %)
No	6 (3.65 %)	2 (1.81 %)	33 (31.73 %)	52 (33.98 %)	8 (2.91 %)	85 (33.07 %)	39 (14.55 %)	54 (20.53 %)	93 (17.51 %)
Not sure/ cannot say	0 (0.00 %)	0 (0.00 %)	12 (11.53 %)	20 (13.07 %)	0 (0.00 %)	32 (12.45 %)	12 (4.47 %)	20 (7.60 %)	32 (6.02 %)
**Self-isolate**									
Yes	160 (97.56 %)	106 (96.36 %)	101 (97.11 %)	142 (92.81 %)	266 (97.08 %)	243 (94.55 %)	261 (97.38 %)	248 (94.29 %)	509 (95.85 %)
No	3 (1.82 %)	4 (3.63 %)	2 (1.92 %)	7 (4.57 %)	7 (2.55 %)	9 (3.50 %)	5 (1.86 %)	11 (4.18 %)	16 (3.01 %)
Not sure/ cannot say	1 (0.60 %)	0 (0.00 %)	1 (0.96 %)	4 (2.61 %)	1 (0.36 %)	5 (1.94 %)	2 (0.74 %)	4 (1.52 %)	6 (1.12 %)
**Identify and warn/call your close contacts**									
Yes	164 (100.00 %)	108 (98.18 %)	102 (98.07 %)	145 (94.77 %)	272 (99.27 %)	247 (96.10 %)	266 (99.25 %)	253 (96.19 %)	519 (97.74 %)
No	0 (0.00 %)	2 (1.81 %)	2 (1.92 %)	4 (2.61 %)	2 (0.72 %)	6 (2.33 %)	2 (0.74 %)	6 (2.28 %)	8 (1.50 %)
Not sure/ cannot say	0 (0.00 %)	0 (0.00 %)	0 (0.00 %)	4 (2.61 %)	0 (0.00 %)	4 (1.55 %)	0 (0.00 %)	4 (1.52 %)	4 (0.75 %)
**Inform your employer**									
Yes	19 (95.00 %)	18 (100.00 %)	62 (98.41 %)	97 (92.38 %)	37 (97.37 %)	159 (94.64 %)	81 (97.59 %)	115 (93.50 %)	196 (95.15 %)
No	1 (5.00 %)	0 (0.00 %)	1 (1.59 %)	7 (6.67 %)	1 (2.63 %)	8 (4.76 %)	2 (2.41 %)	7 (5.69 %)	9 (4.37 %)
Not sure/ cannot say	0 (0.00 %)	0 (0.00 %)	0 (0.00 %)	1 (0.95 %)	0 (0.00 %)	1 (0.60 %)	0 (0.00 %)	1 (0.81 %)	1 (0.49 %)

In the event of a positive COVID-19 self-test, respondents were highly likely (92.84 %) to communicate their results to a clinic or hotline. This willingness was higher among rural respondents (98.54 %) than urban respondents (86.77 %). Rural respondents were more likely (97.08 %) to visit a healthcare provider for post-test counselling compared with urban respondents (54.47 %). Overall, 97.74 % of respondents indicated that they would notify their contacts of a positive self-test result. This willingness was lowest among urban males for both post-test actions.

**Fig. 3:**
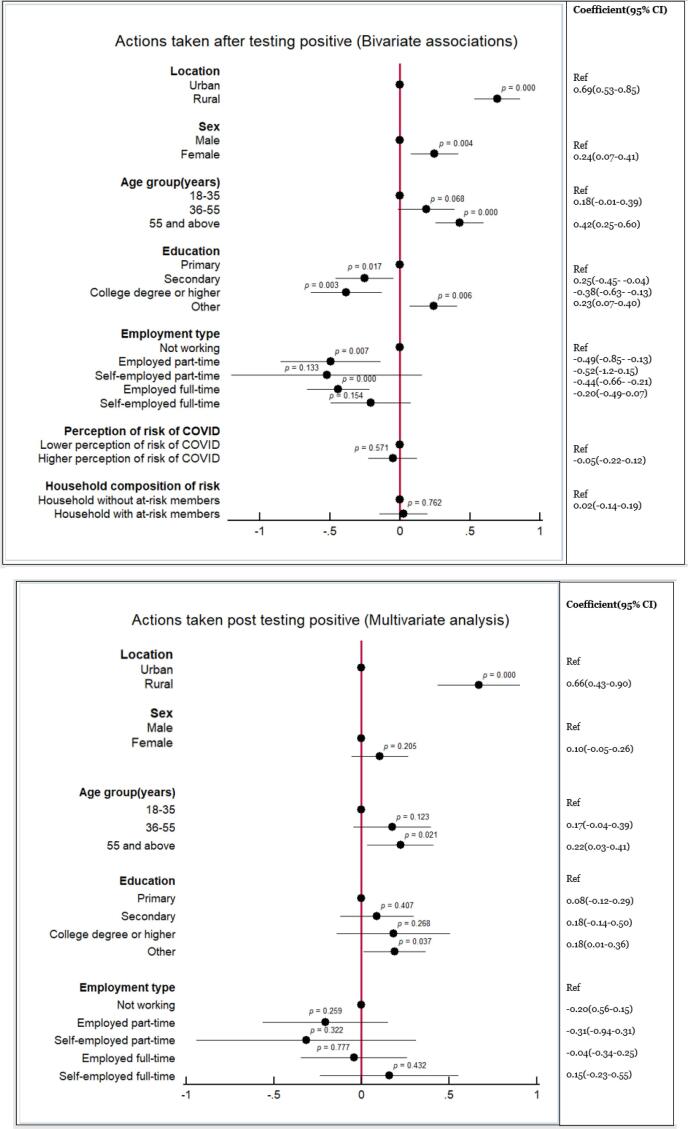
(a) Results of bivariate analysis of the associations of respondents’ characteristics with likelihood to adhere to recommended actions after receiving a positive COVID-19 self-test. (b) Results of multivariate analysis of the associations of respondents’ characteristics with likelihood to adhere to recommended actions after receiving a positive COVID-19 self-test.

The bivariate analyses suggested that respondents from rural areas (coefficient 0.69, 95 %CI: 0.53–0.85, p < 0.000), females (coefficient 0.24, 95 %CI: 0.07–0.41, p = 0.004), those aged ≥ 55 years (coefficient 0.42, 95 %CI: 0.25–0.60, p < 0.000), or with no education (coefficient 0.23, 95 %CI: 0.07–0.40, p = 0.006) would have higher odds of complying with expected actions following a positive self-test (i.e., isolate, communicate results, warn contacts, request counselling) (Fig. 3a). Individuals with secondary (coefficient –0.25, 95 %CI: −0.45 to −0.04, p = 0.017) or college education (coefficient −0.38, 95 %CI: −0.63 to −0.13, p = 0.003) and those employed part- (coefficient −0.49, 95 %CI −0.85 to −0.13, p = 0.007) or full-time (coefficient −0.44, 95 %CI: −0.66 to −0.21, p < 0.000) were less likely to comply with expected actions. The ordinary logistic square regression only confirmed that being a rural respondent (coefficient 0.66, 95 %CI: 0.43–0.90, p < 0.000) and being aged ≥ 55 years (coefficient 0.22, 95 %CI: 0.03–0.41, p = 0.021) were each significantly correlated with willingness to take action (Fig. 3b).

## 4 Discussion

Most participants were supportive of both the general concept of COVID-19 self-testing and of the idea of using a COVID-19 self-test device. These findings aligned with general support for self-testing and decentralized modes of COVID-19 care among individuals in other settings including Indonesia, Kenya, and Nigeria, and support in the South African context surpassed the support observed in other cross-sectional studies regarding COVID-19 self-testing (Manguro et al., 2022, Martínez-Pérez and Schirmer, 2022, Thomas et al., 2022, Goggolidou et al., 2021, Undelikwo et al., 2022). Rural-based South Africans expressed higher likelihood to use self-testing compared with their urban counterparts. Support for COVID-19 self-testing may be related to the accessibility of health facilities, as self-testing eliminates concerns about the time and cost required to access facility-based testing services. However, this study’s findings are limited in terms of assigning meanings to stated likely behaviours. Research should be conducted to assess rural residents’ rationale for opting to self-test and to ascertain the acceptability of novel and point-of-care diagnostics.

It is critical that COVID-19 self-testing, if implemented, is appropriately priced. Pregnancy and HIV self-tests could guide this pricing, given their wide use among South Africans who would be considered resource-poor. Respondents’ willingness to pay, however, does not necessarily reflect their ability to pay for COVID-19 self-testing. At the time of survey administration, unemployment rates averaged 34.9% across rural and urban areas of South Africa and were increasing across nearly all sectors of employment (Statistics South Africa, 2021). Should self-testing become commercially available in South Africa, it may enable vulnerable populations to overcome barriers to health care caused by a shrinking economy, limited labour market, and correspondingly high unemployment rates, but only if self-testing imposes no further risk to employment or income. COVID-19 self-testing should ideally be provided via mechanisms responsive to communities’ socioeconomic circumstances. Further research into ability to pay for COVID-19 self-testing among South Africans is necessary before pricing recommendations can be made.

Rural respondents, for whom the internet can be expensive or difficult to access, would prefer to report COVID-19 test results in-person despite being geographically further from clinics. This may be influenced by greater trust in health services among rural respondents compared with their urban counterparts (Brumwell et al, 2022). Conversely, experiences with long queues in under-resourced urban healthcare facilities may influence urban respondents’ preference for reporting via phone or online (Van Der Hoeven et al., 2012).

Although rural respondents reported a higher willingness to report compared with urban respondents (99.64% versus 79.92%, respectively), rates among both groups were higher than expected, given the socioeconomic risks a positive COVID-19 test could entail. A positive self-test result could lead to periods of unemployment, especially for those already in precarious labour situations (Brumwell et al., 2022, Van Der Hoeven et al., 2012).

Urban respondents’ likelihood to take recommended action upon receiving a positive self-test result was lower for all four explored actions compared with that of their rural counterparts, particularly for seeking in-person post-test counselling. This could reflect less trust in the healthcare system or less access to facility-based care among urban respondents, a higher degree of trust in the healthcare system among rural respondents, or both. Urban areas in South Africa have a long history of protest and expressing political discontent, including with healthcare services. The unwillingness to comply with COVID-19-related regulations made by the state and province governments’ health authorities may reflect the protest culture among urban dwellers and growing dissatisfaction with public management of resources for health (Ngcamu, 2019). Conversely, there may also be a stronger sense of community spirit in less populated rural areas with closely knit communities, influencing individuals to change their hygiene behaviours in agreement with health authorities’ recommendations (Jamieson and van Blerk, 2022). As demonstrated in other African settings, fear of isolation and stigma also mediate individuals’ willingness to report a self-test or take action following a positive COVID-19 self-test (Chabeda et al., 2022, Undelikwo et al., 2022).

As officials seek to integrate self-testing with provider-initiated testing, care delivery, and reporting, it is important to consider the trade-offs between prioritising a population’s strict adherence to reporting and isolation requirements and the ability of decentralised testing methods to contribute to reliable surveillance. While enforcing strict adherence to public health restrictions may enable reliable reporting, it is likely that this approach will also disincentivise the use of self-testing or acting upon a positive self-test result. Considering the impact of lockdowns on South Africans’ economic, physical, and mental health needs, uptake may be low among low-income communities should post-testing requirements conflict with socioeconomic needs (Gittings et al., 2021; Thulare and Moyo, 2021). Public health initiatives must balance the need to understand local epidemics against the need for accessible, affordable self-testing approaches that do not disincentivise appropriate follow-up action.

Self-testing may likely complement decentralised and low-threshold models of testing and reporting that have been developed in settings like South Africa which have a high burden of other infectious diseases like TB and HIV (Winchester & King, 2018). In June 2022, the Africa CDC recommended the use of COVID-19 self-testing as a tool for screening and routine monitoring, particularly for people living with HIV (PLHIV), who are at elevated risk of severe disease and death from COVID-19 (Africa Centres for Disease Control and Prevention, 2022)). Due to their elevated risk, PLHIV benefit from low-threshold forms of testing that facilitate daily or regular use. To support accessible testing for PLHIV and other vulnerable populations, targeted distribution of self-testing kits within health facilities may be paired with distribution via non-profit and community-based organizations that directly serve such populations.

In practice, population-level differences in preferences, characteristics including sociodemographics, and health-seeking behaviours must be considered, should self-testing be integrated with care delivery. Age, gender, and employment status were all associated with likelihood to take recommended actions following a positive self-test. First, older individuals are at higher risk of severe disease and death from COVID-19 and generally have a higher perception of risk; thus, it is unsurprising that advanced age would correlate with willingness to act (Atchison et al., 2021, Eyeberu et al., 2021). Interventions to scale up self-testing must consider strategies that engage younger populations, who are disproportionately affected by unemployment, to encourage necessary post-test action when needed (Galal, 2022).

Second, in South Africa, females tend to demonstrate more proactive health-seeking behaviours compared with males (Leichliter et al., 2011). These disparities may indicate underlying differences in access to care or health status. As suggested through similar studies in Nigeria and Kenya, by reducing the time and cost required for COVID-19 testing, self-testing may promote health-seeking behaviour among males (Chabeda et al., 2022, Undelikwo et al., 2022).

Third, COVID-19 lockdowns in 2020 and 2021 increased economic insecurity among South African communities where unemployment rates were already high, hence employed respondents were less likely to express that they would take action following a positive self-test result (Posel et al., 2021; Thulare and Moyo, 2021). This finding is significant as it may indicate that fears of lost wages or employment prevent individuals from seeking care or notifying contacts about their positive self-test result.

**Limitations**.

Some limitations must be noted. First, our findings reflect a confluence of social, economic, and epidemic conditions at a specific timepoint in South Africa. The study was conducted in September 2021, following the winter peak in COVID-19 cases and significant civil unrest in July 2021, which increased the economic precarity of many communities in Durban, and before the surge in cases driven by the Omicron variant in December 2021. It is unclear whether the seasonality of case rates would affect our findings.

Second, the rural population surveyed comprised a population among whom there is a long history of community engagement, trust-building, and high-quality care and which is dissimilar to the average rural population (Baleta, 2009). Furthermore, the staff who administered the survey in KSD were known to community members, which may have caused some bias in the survey responses. Third, as with all surveys, there is some difficulty interpreting responses and deriving meaningful conclusions relevant to health policy and practice.

Finally, as ethnic identities were self-reported, respondents varied in whether they provided linguistic, national, or ethno-racial identities or refused to answer the question. Due to the large number of variables obtained, statistical analyses were not powered, and this study cannot determine if self-determined national or ethno-racial identities as an independent variable is a predictor of this study’s outcomes.

## 5 Conclusion

Policymakers should consider various factors that may influence the utilisation and utility of COVID-19 self-testing in South Africa. First, self-testing must be affordable or even free for low-income South Africans, and the economic consequences of a positive test should be minimised. However, South Africans in the unregulated work sector may not benefit from improved leave and isolation policies. Second, self-testing must be made accessible, using strategies that are targeted for specific subpopulation. Finally, further research is necessary to understand the reliability of self-testing in South African settings for informing public health surveillance and medical decision-making.

This research provides optimism for self-testing in South Africa, as it is familiar and acceptable to both the rural and urban poor. Appropriate COVID-19 self-testing could result in a better-informed population that can mitigate COVID-19 risks. COVID-19 self-testing may facilitate community participation in public health governance and increase the legitimacy of the health sector, particularly among the urban poor.

## CRediT authorship contribution statement

**Amanda N. Brumwell:** Methodology, Investigation, Writing – original draft, Writing – review & editing, Project administration. **Gbotemi B. Babatunde:** Methodology, Validation, Investigation, Writing – original draft, Writing – review & editing, Project administration. **Michael W. Wilson:** Methodology, Investigation, Resources, Validation, Supervision. **Karl le Roux:** Methodology, Investigation, Resources, Writing – review & editing, Supervision. **Monique M. Marks:** Methodology, Writing – review & editing. **Jamila K. Adam:** Methodology, Writing – review & editing. **Elena Ivanova:** Conceptualization, Methodology. **Deepshikha Batheja:** Methodology, Data curation, Formal analysis, Visualization. **Srishti Goel:** Methodology, Data curation, Formal analysis, Visualization. **Sonjelle Shilton:** Conceptualization, Methodology, Supervision, Funding acquisition. **Guillermo Z. Martínez-Pérez:** Conceptualization, Methodology, Investigation, Supervision, Writing – review & editing.

## Declaration of Competing Interest

The authors declare that they have no known competing financial interests or personal relationships that could have appeared to influence the work reported in this paper.

## Data Availability

Data will be made available on request.
